# Microbiomes of Two Pest Fly Species of Pennsylvania Mushroom Houses

**DOI:** 10.3390/insects15070525

**Published:** 2024-07-12

**Authors:** Joyce M. Sakamoto, Ikkei Shikano, Jason L. Rasgon

**Affiliations:** 1 Department of Entomology, Pennsylvania State University, University Park, PA 16802, USA; jlr54@psu.edu; 2 CTAHR Department of Plant and Environmental Protection Sciences, University of Hawaii at Manoa, Honolulu, HI 96822, USA; ishikano@hawaii.edu; 3 Department of Biochemistry and Molecular Biology, Pennsylvania State University, University Park, PA 16802, USA

**Keywords:** mushroom fly, microbiome, *Lycoriella ingenua*, *Megaselia halterata*, Wolbachia

## Abstract

**Simple Summary:**

Flies inhabit mushrooms or consume them incidentally throughout their lifecycle. In wild conditions, these flies are part of the recycling process and are not considered pests. However, in commercial mushroom houses, these flies can become problematic, as compost pests, through physical damage of mushroom production through consumption, or as vectors of pathogens or nematodes. Here, we describe the bacterial associates of two fly pest species of Pennsylvania mushroom houses.

**Abstract:**

Mushroom cultivation vastly improves the yield of mushrooms under optimized, controlled conditions, but may be susceptible to opportunistic colonization by pest species that can establish themselves, as well as the pathogens and pests they may transmit. Here, we describe our investigation into the bacterial communities of adult *Lycoriella ingenua* (Diptera: Sciaridae) and *Megaselia halterata* (Diptera: Phoridae) collected from button mushroom (*Agaricus bisporus*) production houses in Pennsylvania. We collected adult flies and sequenced the hypervariable v4 region of the bacterial 16S rRNA using the Illumina MiSeq. The most abundant bacterial genus detected in both species was *Wolbachia*, but phylogenetic analysis revealed that the infections are from different clades. Future studies include the characterization of *Wolbachia* infections on fly behavior and biology, comparison of microbial diversity of fly species colonizing wild mushrooms, and other microbiota that may contribute to the success of certain pest fly species.

## 1. Introduction

Fungi are integral components of many ecosystems. Many fungi produce sporocarps, macroscopic structures in which sexual spores develop and from which spores are released. Fungi play many roles in nature: predators, parasites, mutualists, and/or recyclers. A given fungal species may be a mycorrhizal companion of nearby plants, food and shelter for developing invertebrates, and may itself be parasitized by microorganisms while also consuming bacteria and benefiting from bacterial breakdown products. Mycetophagous flies in turn may consume fungal material (mycelia or fruiting bodies) and utilize volatiles from fungal pathogens or bacterial breakdown products to select optimal oviposition sites [1].

Wild sporocarp-forming fungi are populated with a rich diversity of flies [2]. In the northeastern United States, the fruiting structures of the fungal genus *Agaricus* are predominantly colonized by members of the families Drosophilidae, Phoridae, and Tipulidae [3]. In contrast, only a handful of fly species are considered economic pests of mushroom production worldwide. Fly pests are predominantly from the families Phoridae, Sciaridae, and Cecidae [4,5]. In Pennsylvania mushroom farms, the two major pest species are *Lycoriella ingenua* (Dufour 1839, Family Sciaridae) and *Megaselia halterata* (Santos Abreu, 1921, Family Phoridae) [6].

Agricultural monocultures are optimized for maximum crop yield but are also susceptible to opportunistic colonization by pest species that can establish themselves, as well as the pathogens and pests they may transmit. Mushroom houses serve as an ideal experimental environment in which to study the dynamics of microbe–fly–cultivated crop interactions when conditions are optimized for mushroom crop production. Here, we describe our investigation into the bacterial communities of adult *L. ingenua* and *Megaselia halterata* collected from button mushroom (*Agaricus bisporus*) production houses in Pennsylvania.

These two pest species have distinct but overlapping biologies. *L. ingenua* consumes the compost material (and any associated microbes) prior to the addition of mushroom spawn [7]. Once the compost is fully colonized by *Agaricus bisporus*, the populations of *L. ingenua* decline [8]. In contrast, populations of *M. halterata* thrive on mycelial growth, gradually building up from spring until fall and then declining when mushrooms are harvested and beds replaced [9]. We predicted that there would be some overlap in bacterial community composition between the two fly species, but we suspected there would be differences that might be unique to each fly species. We collected specimens of each species, extracted their nucleic acids, and sequenced the bacterial 16S rRNA gene sequences using the Illumina MiSeq sequencing platform. We detected some overlap in bacterial diversity and identified two phylogenetically distinct *Wolbachia* sequences.

## 2. Materials and Methods

### 2.1. Collection Sites and Sampling

We collected *Lycoriella ingenua* and *Megaselia halterata* adults by aspiration from two mushroom production houses in Kennett Square, Chester County, PA, at two times (May and October of 2014; Table 1). Flies were collected in separate vials by species (*L. ingenua* or *M. halterata)* and transported alive on ice to University Park, PA, to prevent damage to the nucleic acids. Flies were then placed at −80 °C until processed.

### 2.2. Sample Processing for Bacterial 16S rRNA Sequencing

To describe the bacterial community composition, we extracted genomic DNA from 40 individual flies (Table 1). DNA extractions were conducted by macerating each adult fly in individual 1.5 mL sterile polypropylene centrifuge tubes using sterile polypropylene pellet pestles in tissue lysing (TL) solution from the Omega E.Z.N.A. Tissue DNA kit (SKU#: D3396, Norcross, GA, USA), following the provided manufacturer’s protocol for extraction from tissue. Extracted nucleic acid samples were submitted for Illumina MiSeq paired-end sequencing of the v4 region of the bacterial 16S rRNA gene. The sequencing facility used the original Caporaso 515F/806R primers [10] (since the 2016 updated EMP primers were not yet available at the time of sequencing in 2015). Sequences (~290 bp) returned from the facility were demultiplexed with primers and adaptors, and barcodes were removed.

### 2.3. Analysis of Bacterial Communities of Flies

Demultiplexed sequences were quality checked, dereplicated, merged (trimmed to 220 bp), filtered to remove chimeric sequences, aligned, and analyzed in RStudio using Dada2 version 1.33.0 and Phyloseq version 1.48.0 and other data visualization tools in several R packages (microbiome 1.26.0, mia 1.12.0, microViz 0.12.3) [11,12,13,14,15]. Reads were assigned taxonomic identity using the Dada2 taxonomy assigner and Silva (v138) reference database of eubacterial 16S ribosomal RNA sequences [11,16,17]. The Amplicon Sequence Variant (ASV) table is provided in the Supplementary Materials. After an initial analysis, we detected taxa that matched mitochondrial sequences or were suspected to be contaminants from other samples sequenced in the same run (see “Post-Illumina sequence confirmation and phylogenetic placement of *Wolbachia* sequences”). Thereafter, we filtered out reads matching “mitochondria”, “*Rickettsia*”, and “*Rickettsiella*” before continuing with diversity analyses. Shannon and inverse Simpson indices were used for measuring richness and evenness. Statistical comparisons within species were performed using the Kruskal–Wallis test. Community dissimilarity (Bray–Curtis index) was evaluated between groups. Principal Coordinate Analyses (PCoA) were performed and plotted to visualize bacterial community structure between groups using Phyloseq. Statistical comparison between groups was performed to run permutational multivariate analyses of variance (PERMANOVA using 999 permutations). The significance level was set to 0.05.

### 2.4. Post-Illumina Sequence Confirmation and Phylogenetic Placement of Wolbachia Sequences

Our samples were sequenced with samples from other arthropod studies (two different mosquito species and a tick species). Because of this, it was important to confirm the presence of the three bacterial genera that were also detected in one or more of those arthropod hosts. We used PCR primers to test for Rickettsia, Rickettsiella, and Wolbachia [18,19]. We did not detect the presence of Rickettsia or Rickettsiella, both of which were taxa in high abundance in tick samples sequenced on the same run. However, we did detect fragments of Wolbachia 16S rRNA using primers W-Specf (5′-CATACCTATTCGAAGGGATAG-3′) and W-Specr (5′-AGCTTCGAGTGAAACCAATTC-3′) to amplify a 438 bp fragment [18]. Amplicons from four fly samples (two of each fly species) were gel-separated, purified, and submitted for Sanger sequencing. Amplicons were aligned to known GenBank deposited sequences of Wolbachia, trimmed to 330 bp to eliminate indels, and phylogenies estimated using Maximum Likelihood with MEGA X using the best-estimated model of evolution selected by jmodeltest [20,21].

## 3. Results

### 3.1. Bacterial Community Composition

**Figure 1:**
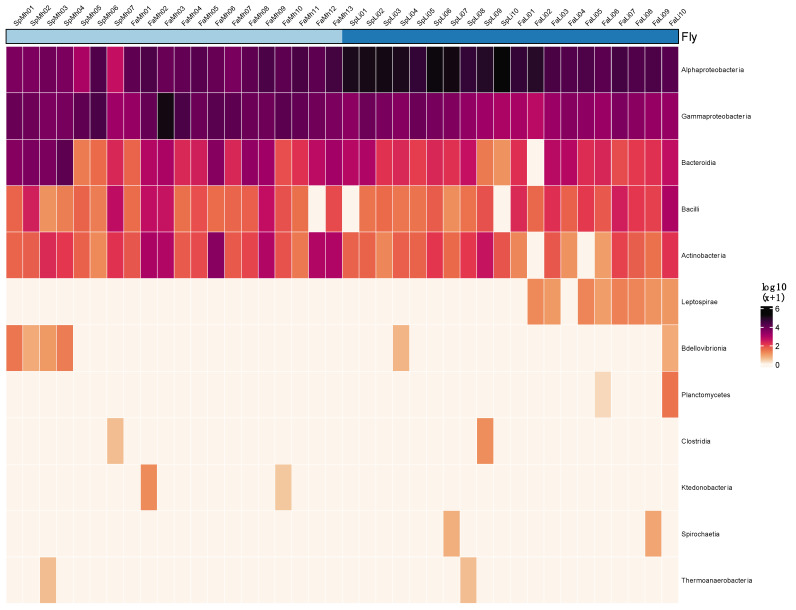
Absolute read counts (Log10(x + 1)) for fly specimens by bacterial Class. Darker colors represent higher reads/taxon. Sample names: samples of *Megaselia halterata* (Phoridae) are denoted by “Mh”, while those of *Lycoriella ingenua* (Sciaridae) are denoted by “Li”. Top horizontal bar denotes fly species: lighter blue represents specimens of *M. halterata*; darker blue represents *L. ingenua.* Heatmap generated using microViz, using the viridis color palette option “rocket” [15].

In total, 2,389,953 16S rRNA reads passed quality control and chimera checking. After the removal of singletons and matches to *Rickettsia*, *Rickettsiella*, and mitochondria, the total number of reads was 2,276,353. Absolute read counts were higher from *L. ingenua* (1,626,951) than from *M. halterata* (685,794) (Figure 1). The five taxa at the Class level that accounted for the majority of the reads were Alpha-proteobacteria, Gamma-Proteobacteria, Bacteroidiia, Bacilli, and Actinobacteria. The 10 most abundant families matched Anaplasmataceae, Enterobacteriaceae, Weeksellaceae, Yersiniaceae, Pseudomonadaceae, Moraxellaceae, Burkholderiaceae, Acetobacteraceae, Rhodobacteraceae, and Aeromonadaceae (Supplementary Figure S1). When examining prevalence across all samples, *Wolbachia* was detected in 95% (38 of 40) of all samples across both species and accounted for 75.8% (*n* = 1,752,473) of total reads. *Wolbachia*, *Serratia* (Yersiniaceae), *Ralstonia* (Burkholderiaceae), *Cedecea* (Enterobacteriaceae), Asaia (Acetobacteriaceae), and *Aeromonas* (Aeromonadaceae) were found in 36 or more of 40 specimens (90% prevalence).

**Figure 2:**
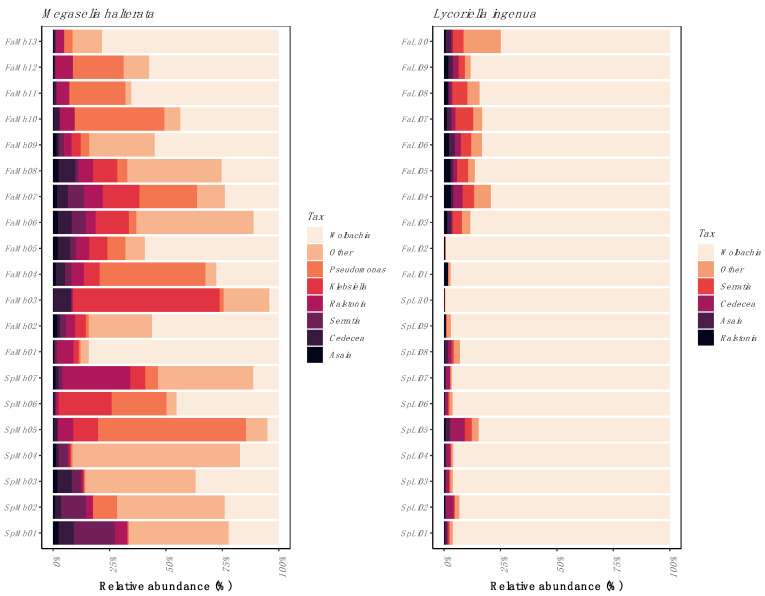
Relative read abundance of bacterial genera by fly species. Relative bacterial read abundance by individuals for each species of fly. Mh = *Megaselia halterata* (Phoridae); Li = *Lycoriella ingenua* (Sciaridae); Fa = fall; S*p* = spring. Rare reads with a prevalence of less than 50% and detection below 0.1% were aggregated into “Other”. Barplot generated using the R package “microbiome” [13].

Relative abundance revealed a marked difference between the two taxa. *Wolbachia* (Class Alpha-Proteobacteria: Family Anaplasmataceae) was the dominant taxon in all individuals of *L. ingenua*, but it was not always the most abundant taxon in *M. halterata* (Figure 2). Gamma-bacteria *Klebsiella* (Enterobacteriaceae) and *Pseudomonas* (Pseudomonadaceae) were often more abundant in *M. halterata* than in *L. ingenua* (Figure 2).

### 3.2. Bacterial Diversity

**Figure 3:**
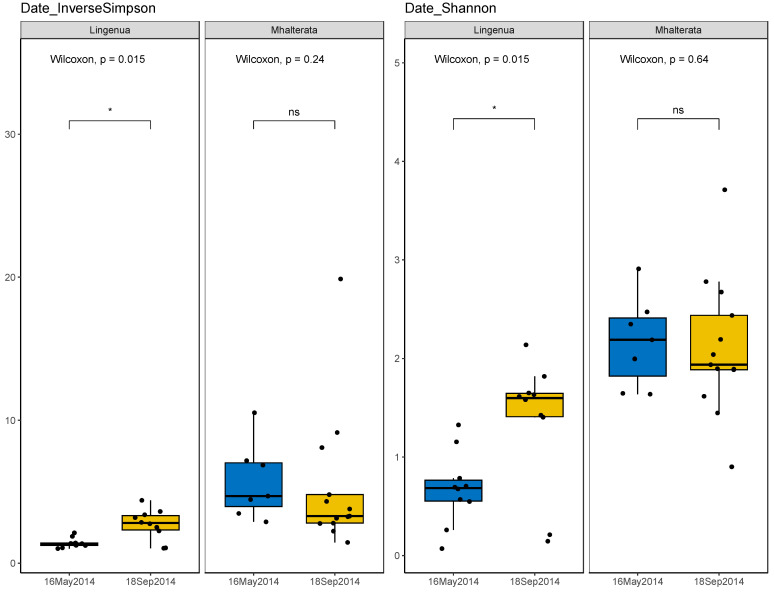
Comparison of alpha diversity by date within each fly species using inverse Simpson and Shannon indices. There was a significant difference in diversity in *L. ingenua* samples collected in May versus September. This difference in diversity was not observed in *M. halterata* between collection dates. Barplot generated using the R package “microbiome” [13].

The *L. ingenua* microbiota was not evenly distributed, and fewer taxa (lower richness) were identified compared to *M. halterata*. We detected significant differences in diversity between collection times in *L. ingenua*, but not in *M. halterata* (Figure 3). While the alpha diversity measures of *L. ingenua* individuals differed between May and September collections, this was not the case for *M. halterata* (most *M. halterata* bacterial taxa clustered together regardless of dates).

**Figure 4:**
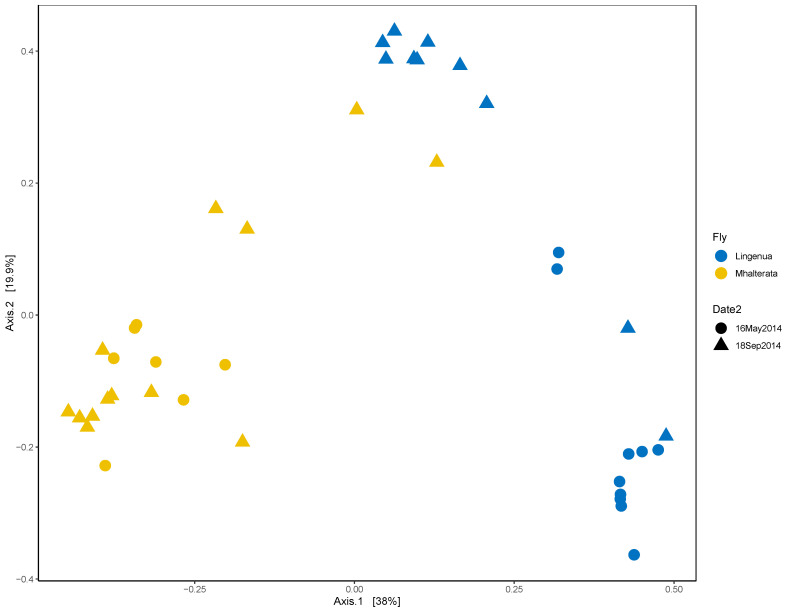
Two-dimensional density plot for both fly species. Data are plotted using a Principal Coordinate Analysis (PCoA) with the Bray–Curtis dissimilarity measure. Samples closer together are more similar in diversity of samples than those that are farther away. Microbial community diversity of *L. ingenua* and *M. halterata* specimens clustered separately in spring-collected specimens, but were more diffuse and overlapped between species in the fall-collected specimens. PCoA plot generated using the R package “microbiome” [13]. Shapes refer to collection dates; colors represent fly species (Circles = 16May2014, Triangles = 18Sept2024, Blue = “*L. ingenua*”, Yellow = “*M. halterata*”*).*

When we examined the beta diversity, we observed that the diversity measures of the two species were distinct from each other, although there was some clustering between fly species that corresponded to the fall collection (Figure 4). We confirmed that there was a significant interaction between fly species and collection date using a PERMANOVA analysis (Species *p* = 0.001; Date *p* = 0.022; Species*Date *p* = 0.008). We therefore analyzed the two species separately to confirm the effect of the collection date. In both species, there was a significant effect of collection date (Li, R2 = 0.36726, *p* = 0.005; Mh, R2 = 0.13511, *p* = 0.003).

### 3.3. Sequence Confirmation and Phylogenetic Placement of Wolbachia Sequences

**Figure 5:**
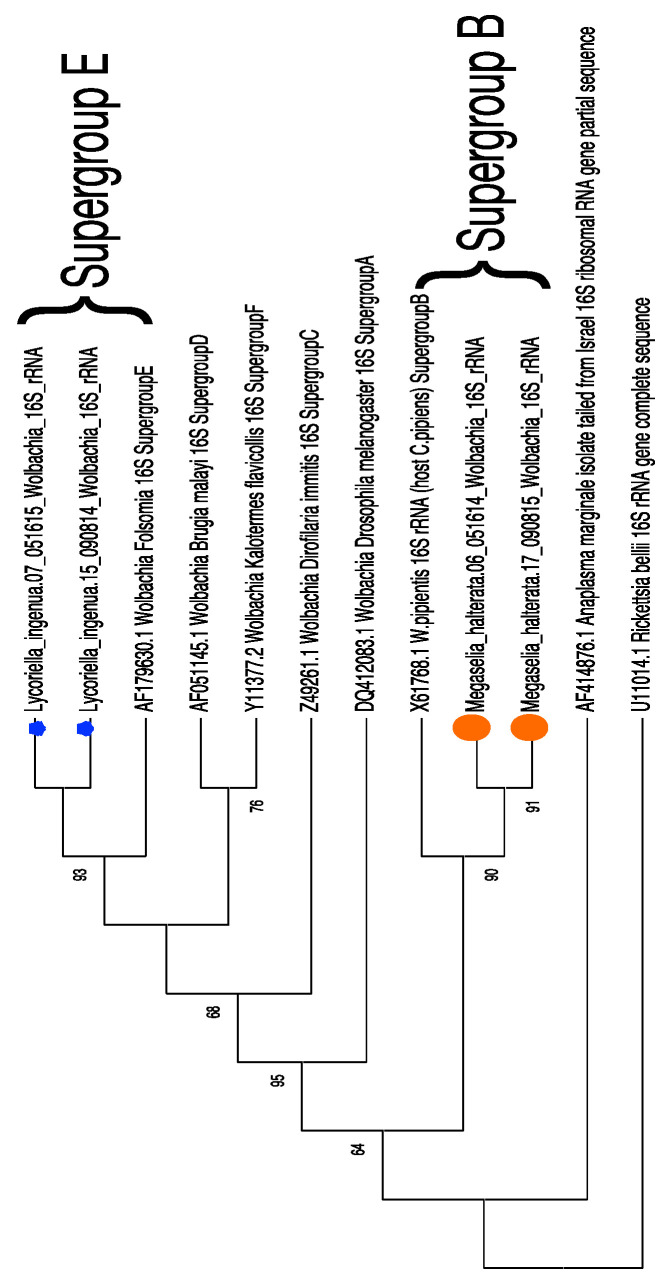
Maximum Likelihood phylogenetic tree of *Wolbachia* 16SrRNA sequences from each fly species. Analyses were conducted using MEGA X [21]. The evolutionary history was inferred using the Maximum Likelihood method and the Tamura–Nei model. The bootstrap consensus tree was inferred from 1000 replicates. Branches with less than 50% bootstrap support are collapsed. Initial trees are obtained by Neighbor-joining and BioNJ algorithms. Evolutionary rate differences among sites were modeled using gamma distribution with the inclusion of some evolutionary invariable sites (+G, +I). All positions with less than 95% coverage were excluded. The trimmed and aligned fragment length was 330 bp. *Anaplasma marginale* and *Rickettsia bellii* were outgroups. Lm07 and Lm15 (stars) = *Lycoriella ingenua*; Mh06 and Mh17 (circles) = *Megaselia halterata.* Supergroups E and B represent *Wolbachia* Supergroups into which the fly *Wolbachia* sequences clustered.

Fragments of *Wolbachia* 16S rRNA sequences from four samples (two from each fly species) were amplified, gel-purified, and submitted for Sanger sequencing. We confirmed that the *Wolbachia* sequences identified in the dataset were not due to sequence contamination and that the isolates from each fly species were from phylogenetically distinct clades (Supergroup E for *L. ingenua* and Supergroup B for *M. halterata*) (Figure 5).

## 4. Discussion

While there have been other microbiome studies in mushroom cultivation settings, they largely focus on the mushrooms and the substrate, on associated fungi, or on viruses [9,22,23,24,25]. In this study, we address the presence of bacteria in two mushroom fly pest species. We observed distinct bacterial community compositions and also observed an effect of collection date, particularly in *L. ingenua.*

We investigated the two collection dates in order to assess whether microbial communities changed between spring and fall populations. The bacterial community composition is highly dynamic and dependent on the ecology of the mushroom house. After the first flush (=crop) of mushrooms, some taxa (e.g., Proteobacteria) decline, but are replaced in abundance by Actinobacteria and Firmicutes [25]. It is therefore conceivable that flies intimately associated with mushroom compost and casing might acquire some of their microbiota, but the extent to which they both harbored the same taxa was not known.

The microbial diversity between the two fly species overlapped somewhat in the fall. This could be explained by their respective biologies. *L. ingenua* is a generalist mycophagous insect, readily consuming the mycelium, colonized compost, mushroom primorida, and all parts of the fully developed sporocarps. In contrast, *M. halterata* is more selective (“oligophagous”), feeding only on actively growing hyphal tips [26]. As the two populations increase over the season from spring to fall, they experience increased competition for resources that become depleted over time, and consequently may acquire or share microbes present in the substrate.

Because bacterial read counts were much higher from *L. ingenua* than from *M. halterata* (Figure 1), it was important to consider this when interpreting differences in microbial communities between fly species. For instance, *Serratia* was found in higher absolute abundance in *L. ingenua*, but this only accounted for proportionally less than 10% of the total taxonomic abundance. The absolute and relative abundances of *Klebsiella* and *Pseudomonas* were higher in *M. halterata* than in *L. ingenua*; the dominant bacterial taxon detected in both fly species was confirmed to be *Wolbachia*.

*Wolbachia* occurred at higher relative (and absolute) abundance in *L. ingenua* than in *M. halterata*. It is not unusual to find *Wolbachia* in fly species. However, phylogenetic analysis suggests that the *Wolbachia* found in both fly species may have been acquired independently. *Wolbachia* sequences contained in the fly species were determined to be from different clades. The presence of *Wolbachia* in both fly species was confirmed (post-Illumina sequencing) because of a concern that the sequences represented contamination from mosquito samples that were also sequenced in the same run. However, while the *M. halterata Wolbachia* was from a similar clade to *Culex pipiens Wolbachia*, it was distinct from the *Wolbachia* sequenced from the mosquito samples. Further, the *Wolbachia* identified in *L. ingenua* was from a completely different cluster (within the Supergroup E clade), a clade that has been previously associated primarily with springtails and several mite species [27,28]. One predatory mite (*Hypoaspis aculeifer*), known to be an effective biocontrol agent against both species of flies, was not observed or known to be in these mushroom houses, but even if it had been present, it is not a species known to harbor *Wolbachia* [7,29]. Since the sequencing was conducted on whole flies (flies were too small to dissect for sufficient DNA for sequencing), we cannot exclude the possibility that the *Wolbachia* detected came from infected springtails or mites that may have been consumed by fly larvae in the mushroom mats. Whether or not the *Wolbachia* found in *L. ingenua* existed in the flies as a co-evolved associate or acquired through horizontal transfer through interactions with other organisms in mushroom beds (e.g., springtails or mites) needs further research.

*Pseudomonas* is a ubiquitous bacterial taxon, and several species of *Pseudomonas* have been described from mushroom farms. Its presence was therefore not a surprise in our sequence data. While some species of *Pseudomonas* are important enhancers of mushroom development (metabolizing compost compounds that might otherwise inhibit *A. bisporus* primordial development), other species (e.g., *P. tolaasii* and *P. reactans*) are known pathogens [22,24]. Although we detected *Pseudomonas* in both fly species, we did not isolate or characterize them and cannot ascribe their nature as pathogenic, beneficial, or commensal within the mushroom house microbiome.

### 4.1. Limitations of the Study

Our study had some limitations that could be addressed in future studies. In some flies (e.g., *Bactrocera tryoni*, the Queensland fruit fly), the microbial communities can shift from immature to adult stages [30]. We are unable to speculate as to the effect the bacterial communities have on larval development or adult behavior because our samples were collected in the same year, albeit from different seasons.

We cannot speculate whether the *Wolbachia* detected in this study caused sex ratio distortion or reproductive effects because we did not separate the males or females, nor did we examine immature life stages. However, since *Wolbachia* infections can be cleared through antibiotic treatment, we could potentially examine the behavior and interactions of *Wolbachia*-free flies with mushroom substrates, other invertebrates, and associated microbiota in future studies.

We do not know the extent to which the microbial communities of flies and mushrooms are shared, how the microbiota influences fly behavior, or the secondary ecological impacts of fly microbiota on parasitoids of mushroom flies, springtails, predatory mites, or nematodes. These are areas that could be explored further in later studies.

### 4.2. Future Studies

Mushroom cultivation began in the 1600s, but structures or caves were not used until the early 1900s [31]. Modern mushroom houses were started in the early 1900s but were quickly plagued by sciarid pests [32]. While earlier attempts at control included chemical applications, the quick development of resistance necessitated changes in cultivation practices and biocontrol agents as integrated pest management strategies [33,34]. Cultivation practices such as compost pasteurization and the use of chemicals and biocontrol agents (e.g., predatory mites, entomopathogenic nematodes and fungi) can help control fly pests, but exclusion is preferred [7,35,36]. It should be noted that pasteurization alone may not be sufficient, as adult female sciarids (*L. ingenua*) are attracted to compost and the volatiles released by pathogenic molds [37].

Cultivated button mushroom farming represents a rich microbial ecosystem under fairly homogeneous environmental conditions. The current study connects one more piece of the multitrophic ecological puzzle, but many questions are still unanswered. For instance, can the interactions and dynamics of mushroom flies with other microbial (e.g., viruses or nematodes) or invertebrate associates be used to facilitate the biocontrol of flies, bacterial pathogens, or fungal pathogens of mushroom houses?

One such study we are currently exploring is the identification of viral communities and the potential for both of these fly species to serve as vectors of mushroom pathogens. In a preliminary viral study of mushroom flies, we identified a putative fungal hypovirus in the spawn (unpublished data). While we did not detect that hypovirus in flies, it is conceivable that (given the polyphagous nature of *L. ingenua)* the fly might serve as a vector and/or reservoir of viral pathogens of fungi. In a study by Liu et al. (2016), a mycovirus of the plant pathogen *Sclerotinia sclerotiorum* (named *Sclerotinia sclerotiorum hypovirulence-associated DNA virus 1*) was shown to infect and replicate in L. ingenua, and to be transovarially transmitted [38].

One of the biological differences between the two fly species is that, while *L. ingenua* is found throughout the growing season, *M. halterata* populations build up from spring until fall, and then decline in winter. *M. halterata* adults leave the mushroom houses in fall to mate and may maintain their populations outside of the houses. However, evidence of phorid presence was not detected in adjacent residential properties [7,9]. One future goal is to identify alternative hosts that may support *M. halterata* or serve as refugia for overwintering. Another is to compare the microbial dynamics between years, since, if *M. halterata* overwinters outside of mushroom houses, it is likely exposed to different microbial pressures and could bring external pathogens back into the mushroom houses the following year.

While the purpose of the exploration of mushroom fly microbial dynamics was to identify biocontrol options in a cultivated setting, a broader ecological question we could not ignore was the following: What are the factors that dictate which fly species becomes a pest? Wild mycophagous flies are dependent on a resource whose abundance is heavily affected by rainfall and other variables. Thus, resource unpredictability would likely favor polyphagy, not host specialization, in mycophagous flies [1]. The diversity of mycophagous fly taxa encountered in wild mushrooms reflects this and represents an arena for resource competition. Wild mushrooms (*Agaricus* spp. in particular) in the northeastern United States are largely colonized by mycophagous drosophilids (*Drosophila* and *Leucophenga* spp.), wood gnats, mushroom flies, and crane flies [1,3]. Although a limited food source (e.g., single basidiocarp) might result in inter- and intraspecies competition and subsequent reduction in size, an effectively inexhaustible food source (such as a mushroom house) would likely favor mushroom flies.

In theory, any mycophagous fly in the vicinity should benefit from such an abundance of resources. In other regions of the USA and worldwide, other fly species are also pestiferous: cecids *Mycophila speyeri* and *Heteropeza pygmaea* can damage mushroom production, while house and stable flies are nuisance pests of compost heaps [5]. Cultivation and control practices have been successful in excluding or eliminating former pests such as mites and springtails in commercial production houses [6,33]. One future study we are interested in is an in-depth investigation of the multitrophic dynamics of fly colonization of wild mushrooms in adjacent wooded areas to identify possible explanations for the exclusion or establishment of other fly species in cultivated settings.

What role *Wolbachia* plays in the lifecycle of either of these fly species is unknown. Further studies would include attempts to cure colonized flies of *Wolbachia* infections and determine whether/how this affects biology, behavior, or pathogen vector competence. We can also determine the population genetics of the *Wolbachia* isolates in mushroom houses and in wild mushroom populations.

## Figures and Tables

**Table 1:** Fly specimen information table. “SampleID” corresponds to the files submitted to Genbank. “Fly” = fly species; “Date” = collection date; “SampleNo” = simplified graph labels. “Mh” and “Li” refer to *M. halterata* and *L. ingenua*, respectively; “Sp” and “Fa” refer to the collection dates 16 May 2014 and 18 September 2014, respectively.

SampleID	Fly 1	Date	SampleNo
C09Ph051614Mh01	*M. halterata*	16 May 2014	MhSp01
C10Ph051614Mh02	*M. halterata*	16 May 2014	MhSp02
C11Ph051614Mh03	*M. halterata*	16 May 2014	MhSp03
C12Ph051614Mh04	*M. halterata*	16 May 2014	MhSp04
D01Ph051614Mh05	*M. halterata*	16 May 2014	MhSp05
D02Ph051614Mh06	*M. halterata*	16 May 2014	MhSp06
D03Ph051614Mh07	*M. halterata*	16 May 2014	MhSp07
D04Ph091814Mh04	*M. halterata*	18 September 2014	MhFa01
D05Ph091814Mh05	*M. halterata*	18 September 2014	MhFa02
D06Ph091814Mh06	*M. halterata*	18 September 2014	MhFa3
D07Ph091814Mh11	*M. halterata*	18 September 2014	MhFa04
D08Ph091814Mh12	*M. halterata*	18 September 2014	MhFa05
D09Ph091814Mh13	*M. halterata*	18 September 2014	MhFa06
D10Ph091814Mh14	*M. halterata*	18 September 2014	MhFa07
D11Ph091814Mh15	*M. halterata*	18 September 2014	MhFa08
D12Ph091814Mh16	*M. halterata*	18 September 2014	MhFa09
E01Ph091814Mh17	*M. halterata*	18 September 2014	MhFa10
E02Ph091814Mh18	*M. halterata*	18 September 2014	MhFa11
E03Ph091814Mh19	*M. halterata*	18 September 2014	MhFa12
E04Ph091814Mh20	*M. halterata*	18 September 2014	MhFa13
E05Sc051614Li01	*L. ingenua*	16 May 2014	LiSp01
E06Sc051614Li02	*L. ingenua*	16 May 2014	LiSp02
E07Sc051614Li03	*L. ingenua*	16 May 2014	LiSp03
E08Sc051614Li04	*L. ingenua*	16 May 2014	LiSp04
E09Sc051614Li05	*L. ingenua*	16 May 2014	LiSp05
E10Sc051614Li06	*L. ingenua*	16 May 2014	LiSp06
E11Sc051614Li07	*L. ingenua*	16 May 2014	LiSp07
E12Sc051614Li08	*L. ingenua*	16 May 2014	LiSp08
F01Sc051614Li09	*L. ingenua*	16 May 2014	LiSp09
F02Sc051614Li10	*L. ingenua*	16 May 2014	LiSp10
F03Sc091814Li01	*L. ingenua*	18 September 2014	LiFa01
F04Sc091814Li02	*L. ingenua*	18 September 2014	LiFa02
F05Sc091814Li03	*L. ingenua*	18 September 2014	LiFa03
F06Sc091814Li04	*L. ingenua*	18 September 2014	LiFa04
F07Sc091814Li05	*L. ingenua*	18 September 2014	LiFa05
F08Sc091814Li06	*L. ingenua*	18 September 2014	LiFa06
F09Sc091814Li07	*L. ingenua*	18 September 2014	LiFa07
F10Sc091814Li08	*L. ingenua*	18 September 2014	LiFa08
F11Sc091814Li09	*L. ingenua*	18 September 2014	LiFa09
F12Sc091814Li10	*L. ingenua*	18 September 2014	LiFa10

1 There were 7 *M. halterata* collected in spring and 13 *M. halterata* collected in fall.

## Data Availability

Sanger sequences of Wolbachia 16S rRNA fragments can be found at Genbank Accession # PP549140-PP549143. Illumina sequences of bacterial 16S rRNA sequences from fly samples are at Genbank under Bioproject Accession #PRJNA1093092.

## Supplementary Materials

The following supporting information can be downloaded at https://www.mdpi.com/article/10.3390/insects15070525/s1: Figure S1: Absolute read counts (Log10(x + 1)) for fly specimens by bacterial family, Table S1: Combined ASV and taxonomy data (prior to removal of contaminant sequences or singletons).
