# Supplementary figures and images for: Microbiomes of Two Pest Fly Species of Pennsylvania Mushroom Houses
Source: Insects. 2024 Jul 12;15(7):525. doi: 10.3390/insects15070525 (PMC11276769; doi:10.3390/insects15070525)

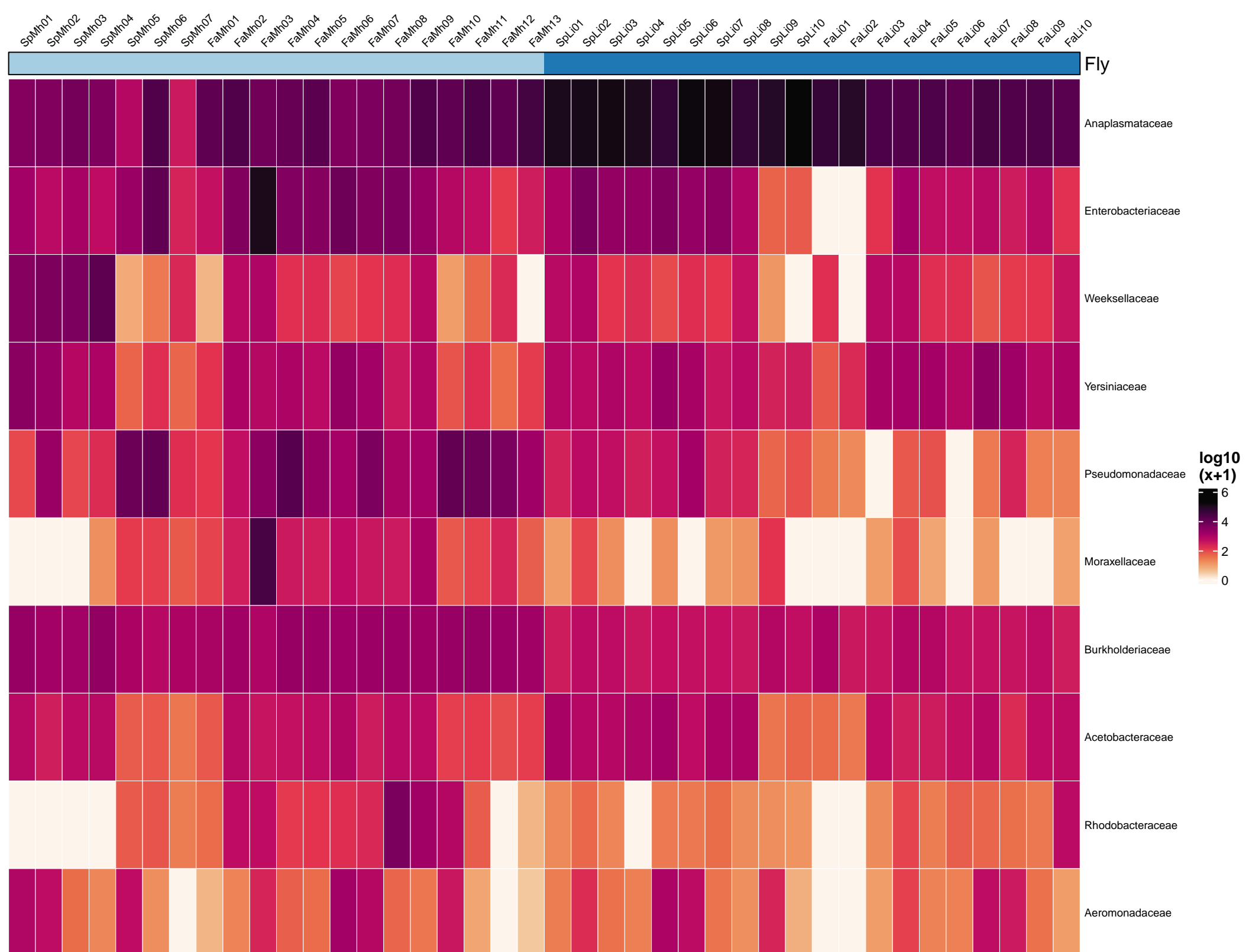

Supplement: Supplementary file 1 [file insects-15-00525-s001.zip › Figure S1. Absolute read counts (Log10(x+1)) for fly specimens by bacterial family.pdf]
